# Interaction of the Morphogenic Protein RodZ with the *Bacillus subtilis* Min System

**DOI:** 10.3389/fmicb.2017.02650

**Published:** 2018-01-18

**Authors:** Katarína Muchová, Zuzana Chromiková, Romana Valenčíková, Imrich Barák

**Affiliations:** Institute of Molecular Biology, Slovak Academy of Sciences, Bratislava, Slovakia

**Keywords:** *Bacillus subtilis*, cell division, cytoskeleton, RodZ, MinJ, protein interactions

## Abstract

Vegetative cell division in *Bacillus subtilis* takes place precisely at the middle of the cell to ensure that two viable daughter cells are formed. The first event in cell division is the positioning of the FtsZ Z-ring at the correct site. This is controlled by the coordinated action of both negative and positive regulators. The existence of positive regulators has been inferred, but none have presently been identified in *B. subtilis*. Noc and the Min system belong to negative regulators; Noc prevents division from occurring over the chromosomes, and the Min system inhibits cell division at the poles. Here we report that the morphogenic protein, RodZ, an essential cell shape determinant, is also required for proper septum positioning during vegetative growth. In *rodZ* mutant cells, the vegetative septum is positioned off center, giving rise to small, round, DNA-containing cells. Searching for the molecular mechanism giving rise to this phenotype led us to discover that RodZ directly interacts with MinJ. We hypothesize that RodZ may aid the Min system in preventing non-medial vegetative division.

## Introduction

Cell division is a complex, highly coordinated process for producing viable progeny. Cell division (cytokinesis) must occur at the right place and at the right time in order to ensure that two daughter cells with a complete DNA complement form. The efficient coordination of chromosome replication, chromosome segregation, and cell division is thus crucial for all dividing cells. In bacteria growing in rich medium, chromosome replication, chromosome segregation, and the assembly of cell division machinery all occur simultaneously (Hajduk et al., 2016). The first event in cell division is the polymerization of the tubulin-like FtsZ into a structure termed the Z-ring at midcell. The Z-ring then recruits over 20 other division proteins to form a divisome (den Blaauwen et al., 2017). In *Bacillus subtilis*, divisome formation takes place in two steps: first, FtsZ assembles early and concomitantly with FtsA, SepF, ZapA, and EzrA; next, after a delay, other division proteins such as FtsL, DivIB, FtsW, Pbp2B and various regulatory proteins (GpsB, DivIVA, MinJ, MinD, and MinC) are recruited to midcell (Gamba et al., 2009; Errington and Wu, 2017).

A key question in bacterial cell division is “How does the cell recognize its midpoint in order to position FtsZ and the division machinery?” The selection of a nascent division site is highly precise and is controlled at several levels. Both positive and negative regulators are involved in this process. Positive regulation is accomplished by factors that promote FtsZ assembly at the correct midcell site, negative regulation includes factors that prevent its assembly close to the cell poles and over the nucleoid (den Blaauwen et al., 2017). In *B. subtilis*, assembly of the DNA replication initiation machinery at the origin of replication is thought to potentiate the midcell for FtsZ assembly (Moriya et al., 2010). It was also shown, that the early cell division protein EzrA contributes to the formation and correct placement of the Z ring in *B. subtilis* (Levin et al., 1999; Chung et al., 2004). However, its role seems to be more complex and EzrA has different functions during cell division (Claessen et al., 2008; Gamba et al., 2015). Marker proteins that are recruited to midcell before FtsZ and promote its assembly at this position have been identified in *Myxococcus xanthus, Streptococcus pneumoni*, and *Streptomyces coelicolor* (Willemse et al., 2011; Treuner-Lange et al., 2013; Fleurie et al., 2014).

The best characterized negative regulators of FtsZ assembly are the Min proteins, which block division at the cell poles, and DNA-associated nucleoid occlusion proteins, which block division in the vicinity of the nucleoid (den Blaauwen et al., 2017).

The *B. subtilis* Min system consists of four proteins: MinC, MinD, DivIVA, and MinJ (Levin et al., 1992; Cha and Stewart, 1997; Edwards and Errington, 1997; Bramkamp et al., 2008; Patrick and Kearns, 2008). MinC is the actual inhibitor: it prevents lateral interactions between FtsZ filaments, thereby inhibiting Z-ring formation (Dajkovic et al., 2008). MinD is a Walker type ATPase that binds reversibly to the membrane and recruits MinC to the membrane, allowing it to interact with FtsZ (de Boer et al., 1991). The MinCD complex is targeted to the cell poles and the division site by MinJ, which interacts with the topological factor DivIVA (Marston et al., 1998; Marston and Errington, 1999; Bramkamp et al., 2008; Patrick and Kearns, 2008). It has been shown that DivIVA has affinity for high negative membrane curvature, which occurs only at invaginating division septa and persists at the cell poles (Lenarcic et al., 2009; Ramamurthi and Losick, 2009; Eswaramoorthy et al., 2011). Soon after the initiation of division, DivIVA and MinJ are recruited to the middle of the cell. MinJ then recruits the MinCD complex, which does not affect ongoing division, but is able to disassemble the divisome as division is completed and does prevent the assembly of a new division complex. Some amount of these proteins must also remain at the completed cell poles to prevent inappropriate minicell division from developing (van Baarle and Bramkamp, 2010).

The cell wall and the cytoskeletal system are the main determinants of cell shape in rod-shaped bacteria. Maintenance of the rod shape is ensured by the coordinated action of two peptidoglycan synthesis mechanisms, one responsible for cell elongation and another for cell division (Young, 2010). Two large protein complexes accomplish the synthesis of peptidoglycan: the divisome acts at the site of division and the elongasome ensures cylindrical growth by inserting peptidoglycan along the long axis of the cell (Szwedziak and Löwe, 2013). In previous work, we demonstrated that the highly conserved membrane protein RodZ is a part of the elongasome and directly interacts with other cytoskeletal proteins, including MreB, Mbl, and MreBH and the morphogenetic proteins MreD and MreC (Muchová et al., 2013). We suggested that RodZ might be part of a multi-protein complex that could help to spatially organize the proteins involved in peptidoglycan synthesis and turnover. We also showed that RodZ is involved in asymmetric cell division and interacts directly with SpoIIE, an essential component of the sporulation septum and a crucial determinant of the activation of σ^F^, the first compartment specific sigma factor, in the forespore (Muchová et al., 2016).

In this study, we report that RodZ is involved in determining the site of vegetative cell division and likely helps to block aberrant non-medial cell division. We demonstrate that RodZ directly interacts with MinJ, a member of Min system. We propose that RodZ can help the Min complex to ensure that the septum forms only at midcell during vegetative growth.

## Materials and methods

### Media and general methods

*Escherichia coli* strains were grown in LB (Ausubel et al., 2001), *B. subtilis* cells were grown in LB, DSM, or SMS/SMM (Spizizzen's minimal salts medium) (Harwood, 1990). When required, media were supplemented with 100 μg ml^−1^ spectinomycin, 10 μg ml^−1^ kanamycin, 5 μg ml^−1^ chloramphenicol, or 1 μg ml^−1^ erythromycin and 25 μg ml^−1^ lincomycin. *P*_*spac*_-driven expression was induced using 0.1 – 1 mM isopropyl β-d-1-thiogalactopyranoside (IPTG); 0.05–0.3% xylose was used to induce *p*_*xyl*_ expression.

Generally, all molecular biology experiments in *B. subtilis* were done using standard protocols (Harwood, 1990).

### Bacterial strains and plasmids

The *B. subtilis* and *E. coli* strains used in this study are shown in Table S1; plasmids used in this study are listed in Table S2; the sequences of the oligonucleotides used in this work are given in Table S3.

To construct pSGrodZ, which carries *mgfp* fused to the 5′-end of *rodZ* under the control of *p*_*xyl*_, a PCR fragment containing the *rodZ* gene was amplified using the rodZSB and rodZEE primers. After digestion with BamHI and EcoRI, the fragment was cloned into a pSG1729 plasmid derivative containing a GFP monomeric mutant (A206K) (Lewis and Marston, 1999; Zacharias et al., 2002).

To replace *minJ* at the native locus with *minJ-ypet*, a PCR fragment containing the cytosolic part of *minJ* (*cyt-minJ*) was digested by EcoRI and KpnI and cloned into the EcoRI and KpnI sites of pX-IIE-Ypet (Muchová et al., 2016), exchanging the *spoIIE* part of the construct. The whole *cyt-minJ-ypet* fusion was excised from the pXminJ-ypet plasmid by EcoRI and PstI digestion and cloned into the EcoRI and PstI sites of pSGIIE-Ypet (Muchová et al., 2016), replacing thus *spoIIE-ypet* fusion. The intermediate construct pXcyt-minJ-ypet as well as the final construct pSG-cyt-minJ-ypet were confirmed by sequencing.

To analyze the interaction of MinJ and RodZ by pull down assay we cloned the cytosolic part of *minJ* and the cytosolic part of *rodZ* in pETDuet-1 (Novagen) that is designed for the co-expression of two target genes. The vector contains two multiple cloning sites, each of which is preceded by a T7 promoter/lac operator and a ribosome binding site. To construct pETminJ-S, a PCR fragment containing the cytosolic part of *minJ* was amplified using the cminJSbg and cminJEX primers and, after digestion with BglII and XhoI, was cloned into a similarly digested pETDuet-1 vector. S-tag (KETAAAKFERQHMDSSTSAA) was added at the C-terminus of the cytosolic part of MinJ, which allows detection of the expressed protein by Western blot analysis using an anti-S tag antibody.

To construct the pETrodZminJ-S plasmid, in which cytosolic part of RodZ is His-tagged, a fragment containing the *rodZ* gene obtained from the BamHI and PstI digestion of pETrodZ was cloned into a pETminJ-S, previously digested with BamHI and PstI.

To express a His-tagged cyt-MinJ for determining the dissociation constant of cyt-RodZ and cyt-MinJ by MicroScale Thermophoresis, pETminJ was constructed. A PCR fragment containing the cytosolic part of the *minJ* gene was prepared using the cminJSB2 and cminJEP primers. To yield pETminJ, this PCR fragment was digested with BamHI and PstI and cloned into a similarly cut pETDuet-1 vector (Novagen).

To analyze the interactions of RodZ and MinJ in *B. subtilis* cells, *cyt-minJ-His* in pSG1151 was constructed. A PCR fragment containing *cyt-minJ-His* was prepared using the cminJEF and cminJHisBR primers with a pSGcyt-minJ-mcherry template. This fragment was cloned into pSGcyt-minJ-mCherry, replacing cyt-minJ-mCherry with cyt-minJ-His.

### Bacterial two-hybrid system and quantitative β-galactosidase assay

Fusions of the *B. subtilis* RodZ, MinJ, MinD, MinC, DivIVA, and Noc proteins to the T25 and T18 fragments of adenylate cyclase were constructed in the BACTH bacterial two hybrid system (Karimova et al., 1998). To test for protein–protein interactions, transformants of *E. coli* BTH101 were plated onto LB plates supplemented with 40 μg ml^−1^ X-Gal (5-bromo-4-chloro-3-indolyl-β-d-galactopyranoside), 0.5 mM IPTG, 100 μg ml^−1^ ampicillin and 30 μg ml^−1^ kanamycin, and grown for 24–48 h at 30°C. β-galactosidase activity was measured as described by Miller (1972) with an extra wash step.

### Protein isolation and purification

*Escherichia coli* BL21 (DE3) strains harboring expression plasmids were grown in LB medium at 37°C. When the OD_600_ of the culture reached 0.5, expression of recombinant proteins was induced by the addition of 1 mM IPTG. After 3 h of further growth at 37°C, the cells were harvested by centrifugation. Cell pellets were resuspended in lysis buffer (20 mM Tris-HCl, pH 8.0, 150 mM NaCl) before being disrupted by sonication. The lysate was centrifuged at 30,000 rpm for 30 min to remove cell debris. His-tagged proteins were purified using a 1 ml Ni Sepharose HP column (GE Healthcare). Proteins were eluted with a 4 ml step-gradient of 40 mM to 1 M imidazole. Co-eluted proteins were identified by Western blot analysis using monoclonal antibodies against the His-tag or the S-tag (Novagen).

For pull down assays from *B. subtilis* cells, 50 ml of IB1536 and IB1659 cultures were harvested after 2.5 h of growth in LB medium. Cells were lysed by sonication in 500 μl of 20 mM Tris-HCl, pH 8, 150 mM NaCl, 1 mg ml^−1^ lysozyme, supplemented with complete protease inhibitors (Roche). Membrane proteins were solubilized with 1% Triton X100 and extracts were incubated with Ni^2+^ Sepharose resin (GE Healthcare) for 1 h at 4°C. Samples were washed thoroughly with 20 mM Tris-HCl, pH 8, 150 mM NaCl, 150 mM imidazole. The resin was then mixed with SDS buffer and boiled to extract the proteins. Western blots were probed with monoclonal anti-His (Biorbyt) and monoclonal anti-GFP antibodies (Sigma Aldrich).

### Microscale thermophoresis

Microscale Thermophoresis was performed as described previously (Muchová et al., 2016). In this experiment, the isolated cytosolic part of MinJ (cyt-MinJ) was fluorescently labeled with the amine-reactive red fluorescent dye NT-647 NHS using the Monolith NT.115 protein labeling kit (NanoTemper Technologies, Germany). The experiment was carried out in 16 capillaries, each of which was filled with reaction buffer containing 34 μM cyt-MinJ and cyt-RodZ at one of a series of concentrations covering a range from 6.7 nM to 221 μM. The relative change of fluorescence in each capillary was plotted as normalized fluorescence, F_norm_, the ratio of fluorescence temperature gradient initialization and fluorescence during heating, which is equivalent to the fraction of bound analyte. A preliminary K_d_ was determined by a nonlinear fitting of the thermophoretic responses to the following equation (Seidel et al., 2013) using NT Analysis (NanoTemper Technologies, Germany).

$$\begin{array}{l} F_{norm}=\frac{\left[A_{0}\right]+\left[T_{0}\right]+K_{d}-\sqrt{{\left(\left[A_{0}\right]+\left[T_{0}\right]+K_{d}\right)}^{2}-4\left[AT\right]}}{2} \end{array}$$

where *F*_*norm*_ = [AT] represents concentration of complexes formed between fluorescent molecules of the analyte [A] and non-fluorescent molecules of the titrant [T], [A_0_] is the known concentration of fluorescent molecule, [T_0_] is the known concentration of the titrant which varies in the capillaries and Kd is the dissociation constant.

### Fluorescence microscopy and image acquisition

Liquid *B. subtilis* cultures were grown in appropriate media as described above. To deplete RodZ, the relevant culture was grown in SMM with 1 mM IPTG for 2 h, diluted to an OD_600_ of 0.05 into a medium lacking IPTG, and incubated for an additional 3 h. Samples were stained with 0.2 μg ml^−1^ DAPI to visualize DNA. For membrane visualization, the fluorescent dye FM 4-64 (Molecular Probes) was used at concentrations of 0.2–1 μg ml^−1^. Cells were examined under the microscope on 1% agarose covered slides. When it was necessary to increase the cell density, cells were concentrated by centrifugation (3 min at 5,000 rpm) and resuspended in a small volume of supernatant prior to examination. All images were obtained with an Olympus BX63 microscope, equipped with a Hamamatsu Orca Camera. Olympus CellP imaging software or Olympus Image-Pro Plus 6.0 were used for image acquisition and analysis.

## Results

### RodZ influences cell division site selection

**Figure 1:**
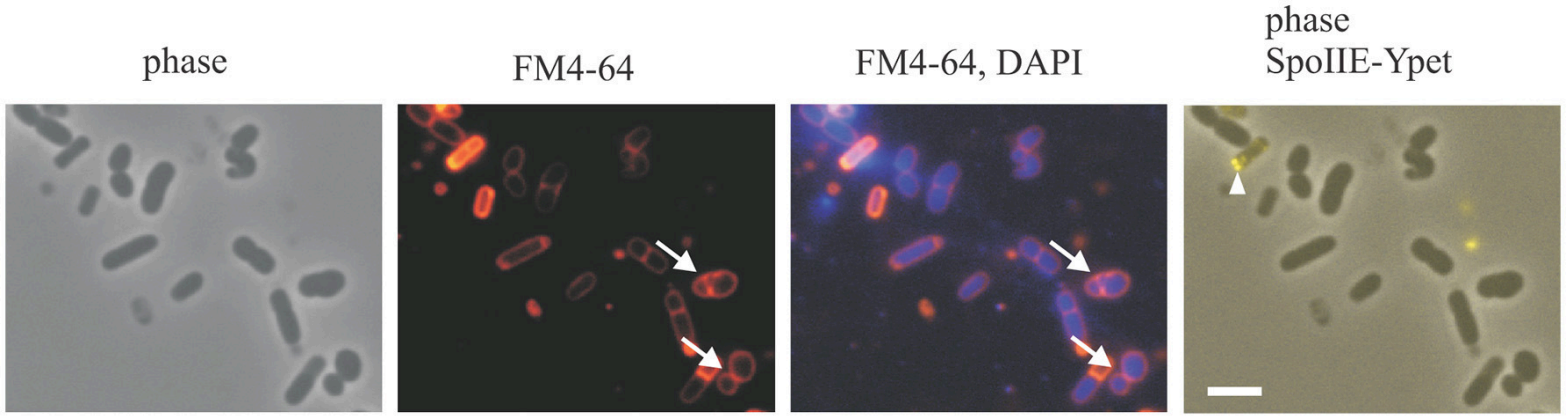
DNA staining of the IB1568 mutant strain. To visualize membranes cells were stained with FM4-64; DNA was visualized with DAPI. The arrows show the locations of asymmetric vegetative septa. The arrowhead shows the position of an asymmetric sporulation septum with a SpoIIE-Ypet signal. The scale bar represents 2 μm.

As mentioned previously strains with disturbed RodZ production produced both wider cells and smaller, round cells resembling the “minicells” that are typical for *min* mutants (Reeve et al., 1973; Muchová et al., 2013). In contrast to the *min* “minicells,” however, these small cells had DNA (Figure 1). DAPI staining showed that 211 from 278 round cells (1457 cells counted) contained DNA. To determine if these small round nucleate cells are the result of vegetative asymmetric cell division, and not sporulation-specific asymmetric division, we analyzed the previously prepared IB1568 strain in which *spoIIE-ypet* is expressed from its native promoter in a background where *rodZ* is under the control of an IPTG-inducible promoter (Muchová et al., 2016). Since SpoIIE is an integral component of the sporulation asymmetric septum, we monitored the formation of asymmetric septa by following the presence of SpoIIE-Ypet in those cells depleted in RodZ. Cells were grown in SMM+salts containing 1 mM IPTG. After 2.5 h of growth, the cells were diluted into a medium lacking IPTG and grown for an additional 3 h. Under these conditions, only a small proportion of cells (8 cells from 1457 cells counted) had already entered sporulation and exhibited a SpoIIE-Ypet signal in their asymmetric septa (Figure 1). Taken together, this suggests that the small round nucleate cells could have arisen by non-medial vegetative cell division. In a control experiment in which the cells were grown in the presence of 1 mM IPTG, 55 cells entered into sporulation and 15 small round nucleate cells were formed (466 cells counted). These results indicate that both unusually low and unusually high levels of RodZ can cause non-medial vegetative septation.

In sum, all our data suggest that RodZ can take part in blocking non-medial division in vegetatively growing cells.

### Bacterial two-hybrid search for RodZ interactions

**Figure 2:**
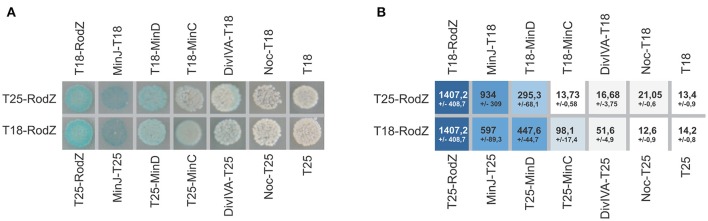
Bacterial two-hybrid analysis of RodZ protein–protein interactions. **(A)** Colonies were spotted onto selective plates containing IPTG and X-Gal. A blue color indicates a positive interaction between each pair of fusion proteins. **(B)** The interactions were quantified using a β-galactosidase assay. The intensity of the blue color indicates the strength of the corresponding positive interaction. Numbers show Miller units of activity and represent the mean ± standard deviation from at least three measurements.

To investigate how RodZ might be involved in division site selection, we searched for RodZ partners among proteins known to control the placement of the division septum. Genes encoding RodZ, MinJ, MinD, MinC, DivIVA, and Noc were cloned in fusion with both domains of adenylate cyclase. The RodZ ORF was mis-annotated in databases and in previous studies (Alyahya et al., 2009; Dempwolff et al., 2011; Muchová et al., 2013, 2016): RodZ actually starts 16 amino acids residues upstream of its annotated position; we used this longer version of RodZ in our experiments. We tested all possible combinations of interacting pairs in assays repeated at least three times (see section Materials and Methods). We found a very strong interaction of RodZ with MinJ, a strong interaction with MinD, and weaker interactions with DivIVA and MinC (Figure 2).

### RodZ interacts with MinJ in pull down assays and microscale thermophoresis

**Figure 3:**
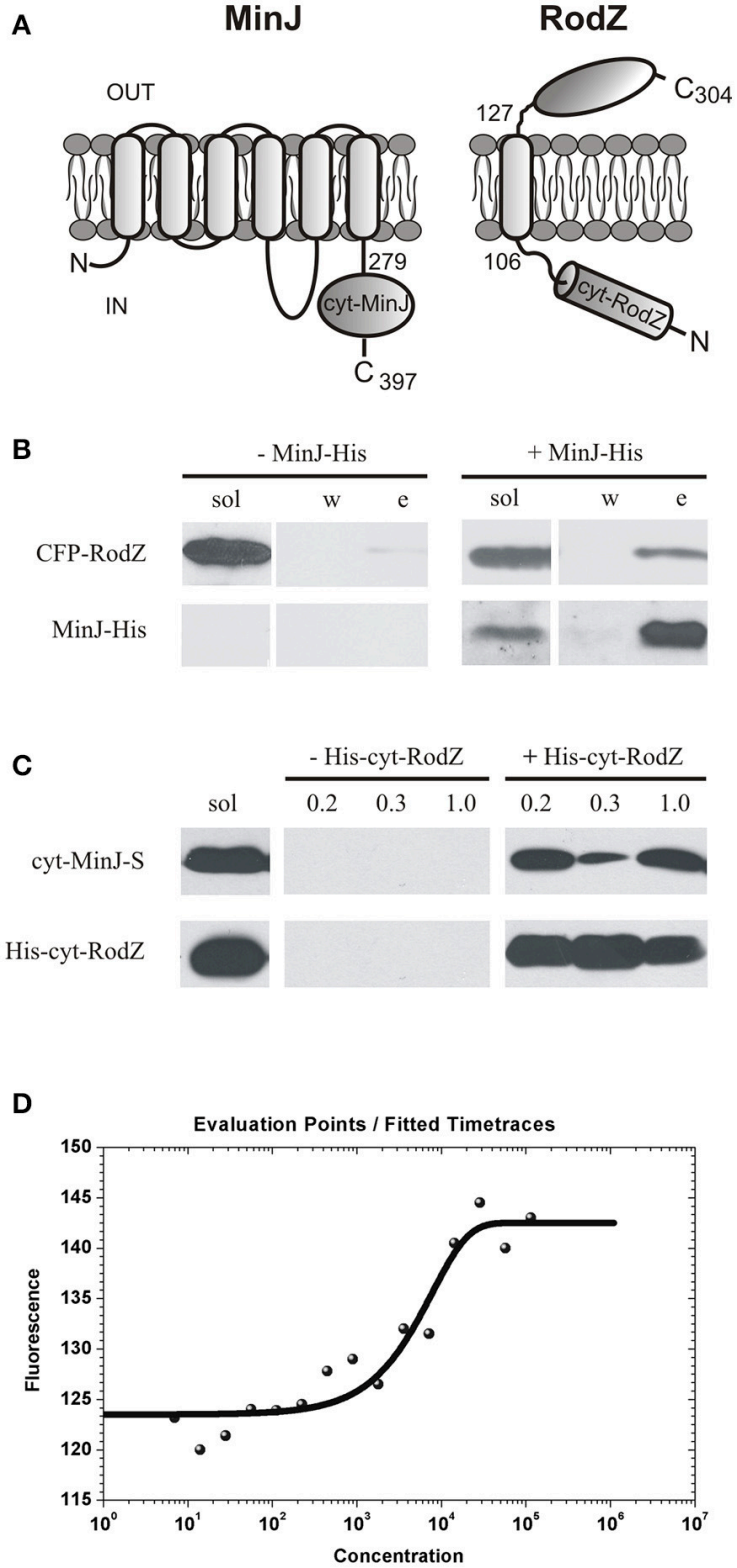
Pull down assay and Microscale Thermophoresis of the RodZ–MinJ interaction. **(A)** Topology of RodZ and MinJ. **(B)** Pull down assay of MinJ and RodZ from *B. subtilis* strains IB1536, producing CFP-RodZ, and IB1659, producing CFP-RodZ and MinJ-His. The pull down assay was performed on Ni Sepharose HP resin. Western blots for detecting CFP-RodZ with an anti-GFP monoclonal antibody and MinJ-His with an anti-His monoclonal antibody are shown. Each Western blot row is from the same exposed blot. Upper row shows CFP-RodZ detected in soluble (sol), wash (w), and elution (e) fractions; lower row shows MinJ-His detected in soluble (sol), wash (w), and elution (e) fractions. CFP-RodZ is not detected in elution fraction when produced alone (–MinJ-His) but is pulled down with His-tagged MinJ (+MinJ-His). **(C)** Pull down assay of proteins isolated from *E. coli* BL21 (DE3). cyt-MinJ was S-tagged while cyt-RodZ was His-tagged. The pull down assay was performed on a Ni Sepharose HP column. Eluted proteins were probed with an anti-S-tag monoclonal antibody (upper row) and an anti-His-tag (lower row) monoclonal antibody by Western blot. Each Western blot row is from the same exposed blot. Both proteins were present in the solubilized extracts (sol). S-tagged cyt-MinJ-S is not detected in elution fractions marked with the (concentrations of imidazole 0.2, 0.3, and 1 M) when produced alone (–His-cyt-RodZ) but is pulled down with His-tagged cyt-RodZ (+His-cyt-RodZ) when the two are co-expressed. **(D)** Microscale Thermophoresis. The relative change of fluorescence in each capillary is expressed as the ratio, in parts per thousand, of the fluorescence before beginning the temperature gradient to the fluorescence detected during temperature gradient. If cyt-RodZ interacts with cyt-MinJ, the ratio changes due to the formation of complexes between the fluorescently labeled analyte (cyt-MinJ) and unlabeled titrant (cyt-RodZ) molecules. The signal obtained during the experiment therefore corresponds directly to the fraction of fluorescently labeled molecules of cyt-MinJ in complex with the cyt-RodZ. Fitting by non-linear least squares to equation stated in section Materials and Methods gave K_d_ = 5.1 ± 0.6 μM.

To confirm that RodZ interacts with MinJ in *B. subtilis*, we prepared strain IB1659 harboring CFP-RodZ and a His- tagged MinJ and performed pull-down assays of RodZ and MinJ from detergent solubilized extracts of these cells. A control experiment used an IB1536 extract producing solely CFP-RodZ. As shown in Figure 3A, MinJ-His bound on a Ni^2+^ resin pulled down CFP-RodZ (Figure 3B), indicating that RodZ interacts with MinJ.

We also performed a cyt-RodZ and cyt-MinJ pull-down assay, using proteins expressed and purified from *E. coli*. We used the pETDuet system to co-express cyt-RodZ with the C-terminal, cytosolic part of MinJ, cyt-MinJ. cyt-RodZ carried a hexa-histidine tag, allowing it to be affinity purified on a Ni^2+^ column; an interacting cyt-MinJ could then be pulled down and subsequently detected using a fused S-tag. SDS-PAGE confirmed that both His-cyt-RodZ and cyt-MinJ-S were expressed in *E. coli*. The proteins were analyzed by Western blotting after purification on a Ni^2+^ column. The results, shown in Figure 3C, demonstrate that S-tagged cyt-MinJ is not detected in elution fractions when produced alone, but is pulled down with His-tagged cyt-RodZ when the two are co-expressed. This result suggests that the cytosolic part of *B. subtilis* RodZ associates with the cytosolic part of MinJ.

Using Microscale Thermophoresis (Figure 3D), we further analyzed the formation of a cyt-RodZ–cyt-MinJ complex. Both proteins were purified on Ni^2+^ column to at least 90% purity as judged by SDS PAGE. In this analysis, a His-tagged cyt-MinJ (see section Material and Methods) was fluorescently labeled and titrated with successive additions of cyt-RodZ. The resulting thermophoresis signals were plotted against cyt-RodZ concentration. Increasing concentrations of cyt-RodZ resulted in increases in the normalized fluorescence, reflecting the altered thermophoretic movement of the fluorescently labeled cyt-MinJ, thereby indicating that ever more molecules of cyt-MinJ were engaged forming in a complex with cyt-RodZ. From the binding curve, we determined that the dissociation constant K_d_ was 5.1 ± 0.6 μM (see section Materials and Methods).

### Co-localization of CFP-RodZ and MinJ-Ypet

**Figure 4:**
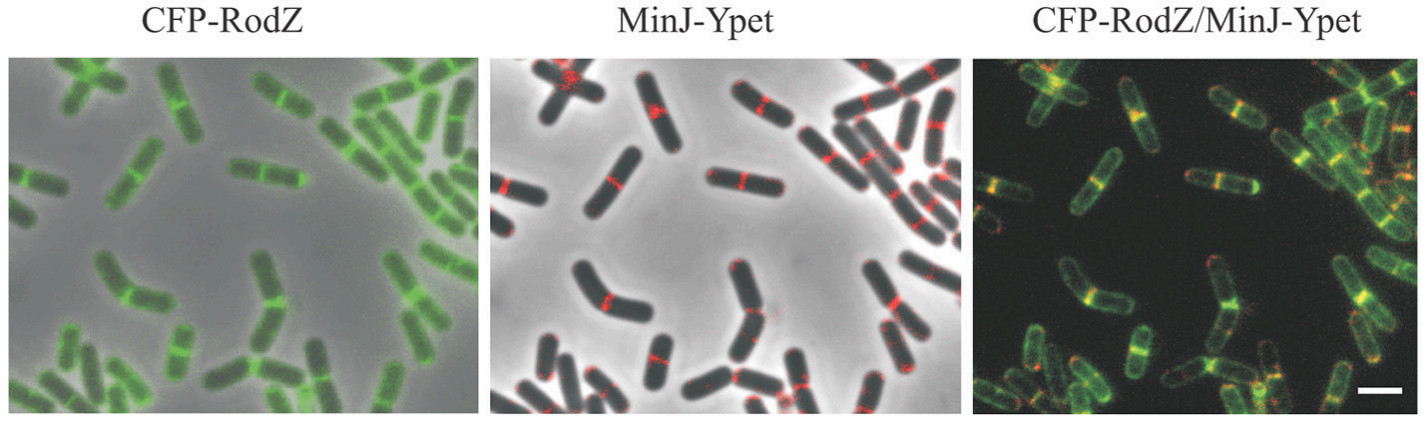
Localization of MinJ and RodZ. Localization of MinJ-Ypet and CFP-RodZ (IB1540) in vegetatively growing cells. Cells were harvested after 3 h of growth. Panel CFP-RodZ shows the localization of CFP-RodZ; panel MinJ-Ypet shows the localization of MinJ-Ypet (MinJ-Ypet signal has been false-colored red); panel CFP-RodZ/MinJ-Ypet shows overlay of images CFP-RodZ and MinJ-Ypet (MinJ-Ypet signal has been false-colored red). The scale bar represents 2 μm.

As shown previously, in addition to helical patches within the membrane, GFP-RodZ also localizes at the midcell septation site and at the cell poles in vegetatively growing cells (Muchová et al., 2013). The localization of RodZ at division septa and the cell poles is reminiscent of that of MinJ (Bramkamp et al., 2008). To study the possible co-localization of MinJ and RodZ, we prepared strain IB1540, in which MinJ is in fusion with Ypet, expressed under the control of its native promoter, and CFP-RodZ is expressed under the control of a xylose inducible promoter at an ectopic *lacA* locus. We observed that both proteins accumulate at division septa and cell poles (Figure 4) during vegetative growth and that they co-localize at these sites. Co-localization was observed in 445 cells from total 500 cells counted.

### RodZ localization in division mutant strains

**Figure 5:**
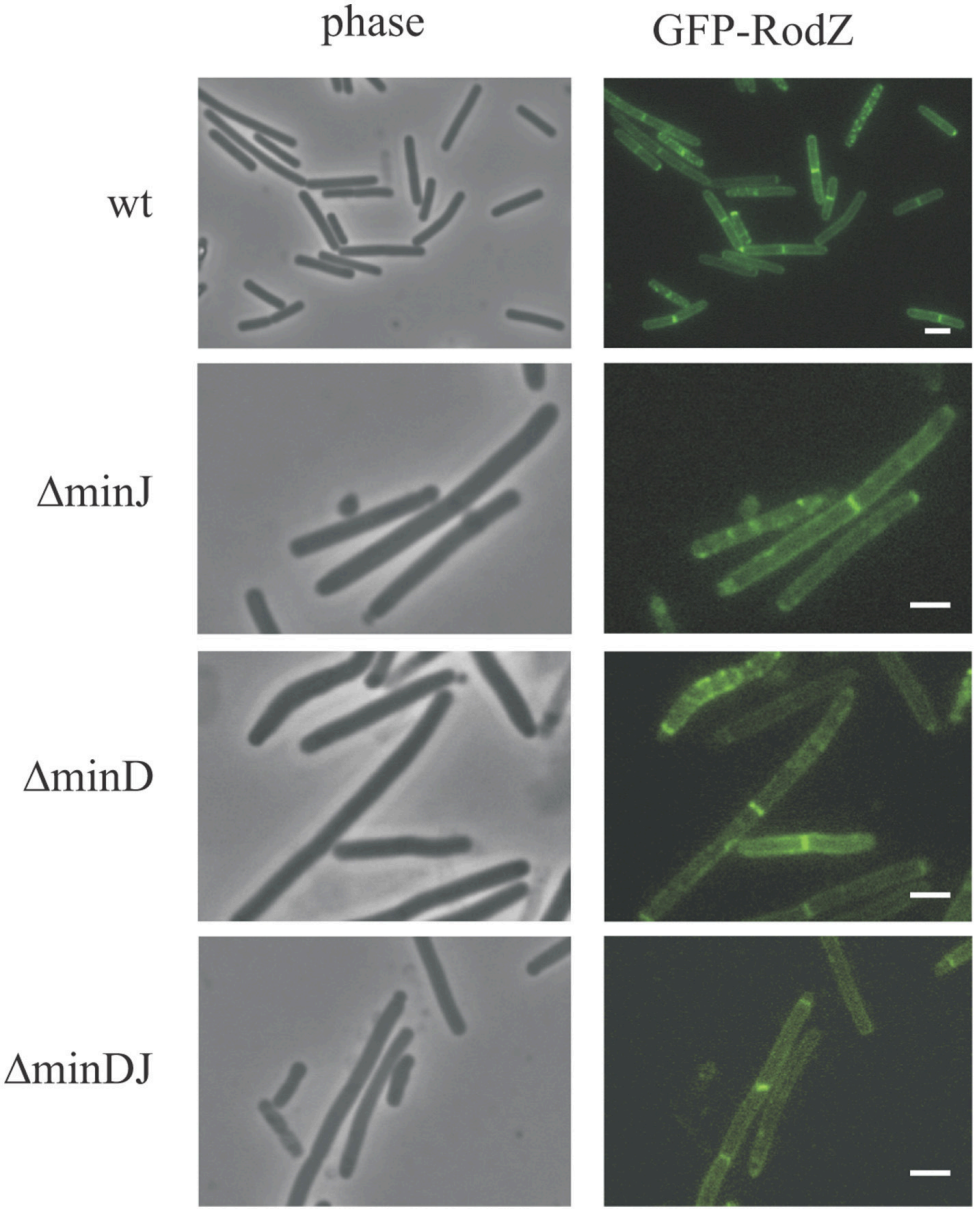
Localization of RodZ in division mutant strains. Localization of GFP-RodZ in Δ*minJ*, Δ*minD*, and Δ*minDJ* strains (IB1657, IB1656, IB1658). Cells were examined after 3 h of growth. The scale bar represents 2 μm.

To test if RodZ localization is dependent on the divisome assembly, we prepared a *minJ* null strain, in which GFP-RodZ is expressed under the control of a xylose-inducible promoter (IB1657). In the absence of MinJ, the divisome does not properly assemble and cells grow as long filaments with rare division septa and occasional minicells (Bramkamp et al., 2008; Patrick and Kearns, 2008). In this background, we observed that GFP-RodZ localizes similarly as in wild type cells (Figure 5). In wild type strain, GFP-RodZ signal was localized in 42 of all septa (total septa 46); in *minJ* null strain, GFP-RodZ was found in 100 septa (total septa 103). To suppress the elongation phenotype of *minJ* deletion (Bramkamp et al., 2008), we constructed a strain harboring an additional *minD* deletion. In this Δ*minJD* strain (IB1658), we found that GFP-RodZ also localizes similarly as in wild type cells (Figure 5); GFP-RodZ was observed in 79 of all septa (total septa 85). It seems, therefore, that RodZ localization is not disturbed in those cells lacking the Min proteins.

### Localization of MinJ is independent of RodZ

**Figure 6:**
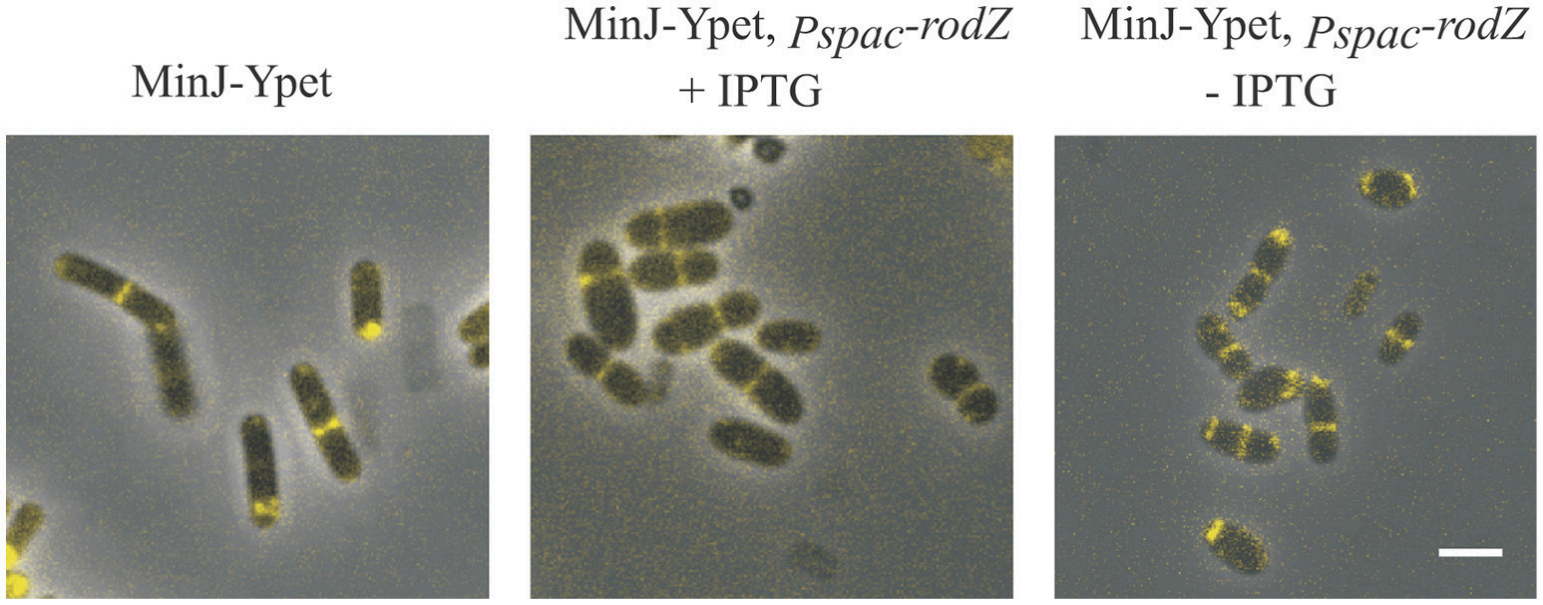
Localization of MinJ in a *rodZ* mutant strain. An IB1570 cell culture was grown in SMM containing 1 mM IPTG, then diluted into a medium lacking IPTG and growth for an additional 3 h. Panel MinJ-Ypet shows the localization of MinJ-Ypet in a wild type background; MinJ-Ypet, *p*_*spac*_*-rodZ* + IPTG shows the localization of MinJ-Ypet in IB1570 grown in the presence of IPTG; MinJ-Ypet, *p*_*spac*_*-rodZ*–IPTG shows localization of MinJ-Ypet in IB1570 grown in the absence of IPTG. The scale bar represents 2 μm.

Our previous results showed that cells whose RodZ production is disturbed are wider and rounder than wild type cells, and that this phenotype is more striking when the cells are grown in SMM minimal medium (Muchová et al., 2013). Since we could not prepare a *rodZ* null mutant (Muchová et al., 2013), we analyzed MinJ localization under conditions of RodZ depletion. For this purpose, we prepared strain IB1570, expressing *minJ-ypet* under the control of its native promoter and *rodZ* under the control of an IPTG-inducible promoter. Cells were grown in SMM containing 1 mM IPTG. After 2.5 h of growth, cells were diluted into a medium lacking IPTG and grown for an additional 3 h. In cells grown either without or with IPTG the MinJ-Ypet signal localized similarly as in wild type cells (Figure 6). These results together indicate that depletion of RodZ does not greatly affect MinJ localization.

To further characterize the possible biological role of RodZ and MinJ interaction, we also prepared a strain (IB1691) in which disruption of *minJ* was introduced into depletion strain (IB1458) where *rodZ* is under the control of an IPTG-inducible promoter. Cells were cultivated as described above. We observed filamentous cells and anucleate minicells specific for absence of MinJ as well as rounder, wider cells and small round DNA containing cells typical for strain depleted of RodZ (not shown). Taken together, phenotype of IB1691 seems to be a combination of the phenotypes of single mutant strains.

## Discussion

Bacteria, like other organisms, proliferate by cell division. A key event in bacterial cell division is the positioning of the Z-ring in the cell. In *B. subtilis*, the Z-ring is anchored to the cytoplasmic membrane via the actin-like protein FtsA together with SepF. This dynamic structure then recruits other proteins required for peptidoglycan synthesis and cytokinesis (den Blaauwen et al., 2017). The Z-ring must be positioned precisely in the middle of the cell to ensure that two equal, viable daughter cells are formed. Despite extensive research, how this is achieved is still not well understood (Monahan and Harry, 2013).

After analyzing *rodZ* mutant strains, we found that both wider cells and smaller, round-shaped cells are formed (Muchová et al., 2013). Suprisingly, these cells contain DNA and thus differ from typical anucleate “minicells” that form as a result of mutations to some cell division genes, which cause the division septum to be placed near a cell pole (Reeve et al., 1973). The small round DNA-containing cells observed in *rodZ* mutants resemble those found in *mreBHA41S* mutant cells, which form both differently shaped cells and round cells containing DNA. Since similar phenotypes were not observed in any *mreB* or *mbl* mutant strains, it seems that MreBH, in addition to cell shape maintenance, also influences cell division (Soufo and Graumann, 2010). Similarly, the presence of small, round nucleate cells in *rodZ* mutants suggests that the RodZ cytoskeletal protein also has a role in cell division site selection. As these types of cells often arise from division that occurs near the cell pole, we investigated whether the septum formed is an asymmetric vegetative septum or asymmetric sporulation septum. Since we did not observe a signal from SpoIIE-Ypet in these cells, we concluded that these cells did not arise by asymmetric sporulation division, but most likely from vegetative division occurring at places other than the midcell. We therefore suggest that RodZ might be involved in inhibiting such non-medial cell division during vegetative growth.

The small, round nucleate cells arising from *rodZ* mutations differ from the recently described “dwarf cells,” small, round DNA containing cells, which can arise from wild-type cells after long growth in minimal medium (Defeu Soufo, 2016). The formation of these “dwarf cells” required Spo0A and SpoIIE-GFP was present in the septum of the majority, however, neither σ^E^ nor σ^F^ were active during their formation. It was therefore suggested that those cells producing the “dwarf cells” had initiated sporulation, but that this process had been aborted, producing shrunken, but otherwise apparently normal, cells. Interestingly, after the addition of fresh medium, they resume growth. Whether the small, round cells formed by *rodZ* mutants can resume growth remains to be investigated.

As mentioned above, the DNA-containing, small *rodZ* mutant cells arise from vegetative cell division when the septum can be formed anywhere between the two chromosomes; normally, the vegetative division septum between the replicated and segregated chromosomes forms precisely at midcell (Barák and Wilkinson, 2007). To examine how RodZ might help to block non-medial division, we searched for RodZ partners among the known negative regulators of division septum positioning: Noc and the Min system proteins. While Noc prevents division from taking place over the chromosome, the Min proteins inhibit division in the nucleoid-free regions at the old and nascent cell poles. Our bacterial two-hybrid analysis revealed that RodZ does not interact with Noc, the main player in nucleoid occlusion, but does interact with the Min proteins, and especially strongly with MinJ.

**Figure 7:**
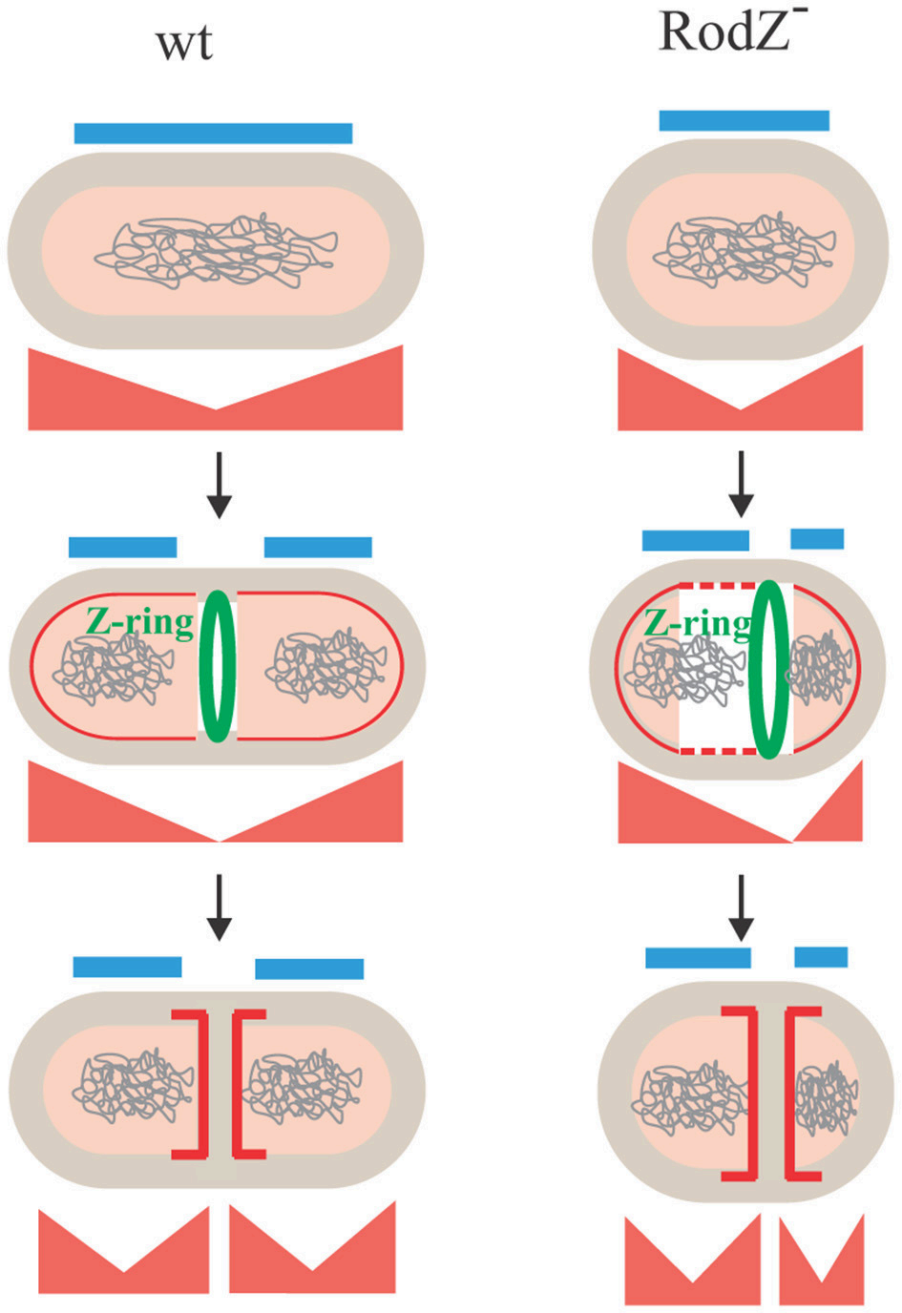
A model of RodZ function in vegetative growth. The horizontal blue lines represent nucleoid occlusion, the Min system is drawn in red, and the red elongated triangles below each cell correspond to the position and concentration of the Min proteins. Early in the cell cycle, Z-ring formation at the midcell is blocked by nucleoid occlusion (blue lines) in both the wild type (wt) and *rodZ* depletion strain IB1458 (RodZ^−^) cells. In wild-type cells, the midcell becomes free for Z-ring assembly after segregation of the replicated chromosomes. The Min system ensures the efficient and proper utilization of the midcell as the only division site. Later, after commitment to division by the recruitment of late division proteins, the Min system is targeted to the septum to allow disassembly of the divisome and to prevent its reassembly (Bramkamp et al., 2008). In *rodZ* mutant cells, after chromosome segregation the absence or an elevated level of RodZ leads to disruption of the Min system's function, allowing the Z-ring to form anywhere between chromosomes. The Min system is later recruited to the septum similarly as in the wild type.

These interactions indicate that RodZ, besides being a component of the elongation machinery (Muchová et al., 2013), could be directly involved in control of division septum positioning. Interestingly, RodZ interacts with many different proteins, including elongasome proteins MreB, Mbl, MreBH, MreC, and MreD (Muchová et al., 2013), sporulation specific SpoIIE (Muchová et al., 2016), and, as shown here, Min proteins. These interactions are likely to be both spatially and cell cycle dependent. During cell elongation, RodZ is a part of the elongasome and probably takes part in the synthesis of peptidoglycan along the lateral cell walls. During cell division, RodZ together with the Min proteins might prevent aberrant septum formation at the midcell region adjacent to the recently formed septum, thereby ensuring that only one Z-ring is formed precisely at the midcell between the replicated and segregated chromosomes (Figure 7). During sporulation, RodZ interacts with SpoIIE and is involved in asymmetric septum formation, stabilization of SpoIIE in the septum and σ^F^ activation (Muchová et al., 2016).

To explore the possible relationship between RodZ and the Min system, we examined the localization of RodZ and MinJ. Comparing the localizations of RodZ and MinJ revealed that these two proteins co-localize at the septum and cell poles. Co-localization of both proteins suggests that RodZ might stabilize the Min complex at these positions, thereby preventing division near the nascent septum and at the poles. We suggest that RodZ, which is a membrane protein, could help to anchor this complex to the site of septum formation through its interaction with MinJ.

Further analysis of RodZ's localization showed that it is not perturbed by *minJ* deletion. In *minJ* mutant cells, cell division arrest occurs after Z-ring formation; it also appears that the MinCD inhibitor acts mainly after Z-ring assembly, preventing the recruitment of the late division proteins (Bramkamp et al., 2008). Localization studies of the late division proteins in *min* mutant strains demonstrated that these proteins fail to disassemble and remain associated with the new pole. The retained divisome can then start new round of cell division leading to the formation of “minicells” (van Baarle and Bramkamp, 2010). Because there were no changes in the RodZ localization pattern in *min* mutant strains, it seems that the Min proteins do not directly determine RodZ's localization. We showed previously that the localization and stability of RodZ depends on the cytoskeletal protein MreB. However, in the presence of higher concentrations of magnesium RodZ also becomes stabilized and localizes properly even in a *mreB* mutant strain (Muchová et al., 2013).

Examining MinJ localization under RodZ depletion conditions revealed that RodZ does not affect MinJ localization. However, in this case, we must also consider that some RodZ might still be present in a depletion strain; even a relatively small amount of RodZ might be sufficient for correct MinJ localization. On the other hand, it is also known that localization of MinJ to the site of septation and cell poles fully depends on DivIVA (Bramkamp et al., 2008), while it is presently not clear what targets RodZ to the vegetative septa and cell poles. It has been found that in *Caulobacter crescentus*, RodZ initially co-localizes with FtsZ when division begins, but then leaves the Z-ring before cell separation (Alyahya et al., 2009). As described above, the localizations of MinJ and RodZ to the division septa and cell poles seem to be mutually independent. Nevertheless, RodZ might still have a role in stabilizing the Min system at these sites and its absence might lead to non-medial cell division. It is also possible that RodZ is important at an earlier step of cell division, before the Min system localizes. This would mean that its absence could allow Z-ring formation to begin anywhere away from the midcell site. In addition, we cannot exclude that the rod shape loss in *rodZ* mutant cells could indirectly affect Z-ring positioning.

In conclusion, our data suggest that RodZ could take part in inhibiting inappropriate non-medial cell division during vegetative growth and in preserving cell division fidelity. Alternatively, RodZ might contribute to the efficient utilization of the midcell for Z-ring formation. In addition, we have also shown that RodZ could fulfill this role through an interaction with MinJ. However, we cannot exclude other, presently unknown, factors and mechanisms, which might be involved in this process.

## Author contributions

KM and IB carried out most of the experimental work. ZC carried out the bacterial two hybrid analysis. RV carried out *B. subtilis* pull down. IB and KM designed the project and wrote the manuscript.

### Conflict of interest statement

The authors declare that the research was conducted in the absence of any commercial or financial relationships that could be construed as a potential conflict of interest.
