# Supplementary material for: Interaction of the Morphogenic Protein RodZ with the Bacillus subtilis Min System
Source: Front Microbiol. 2018 Jan 18;8:2650. doi: 10.3389/fmicb.2017.02650 (PMC5778138; doi:10.3389/fmicb.2017.02650)
Supplement: Supplementary file 1 [file Table1.DOCX]

**Table S1. Bacterial strains**

| **Strain** | **Genotype** | **Source/reference** |
| --- | --- | --- |
| *B. subtilis* |  |  |
| PY79 | Prototrophic derivative of *B. subtilis* 168 | (Youngman et al., 1984) |
| MO1099 | *amy::mls* | (Guérout-Fleury et al., 1996) |
| IB1458 | PY79 *rodZ::pMrodZ* *msl* | (Muchová et al., 2013) |
| IB1626 | MO1099 *amyE::P_xyl_-mgfprodZ spc* | this work |
| IB1056 | *minD::cat* | (Barák et al., 2008) |
| IB1362 | *minJ::kan* | (Jamroskovic et al., 2012) |
| IB1656 | *minD::cat amy::P_xyl_-mgfprodZ spc* | this work |
| IB1657 | *minJ::kan amy::P_xyl_-mgfprodZ spc* | this work |
| IB1658 | *minD::cat minJ::kan P_xyl_-mgfprodZ spc* | this work |
| IB1537 | *p_spoIIE_ spoIIE-ypet cat* | (Muchová et al., 2016) |
| IB1568 | *p_spoIIE_-spoIIEypet cat rodZ::pMrodZ msl* | (Muchová et al., 2016) |
| IB1536 | *lacA::p_xyl_-cfprodZ mls* | (Muchová et al., 2016) |
| IB1539 | *P_minJ_ minJ-ypet* | this work |
| IB1540 | *P_minJ_-minJypet cat lacA::p_xyl_-cfp-rodZ mls* | this work |
| IB1570 | *P_minJ_-minJypet cat rodZ::pMrodZ mls* | this work |
| IB1659 | *P_minJ_-minJHis cat amyE::P_xyl_-cfpprodZ mls* | this work |
| IB1691 | PY79 *rodZ::pMrodZ* *msl minJ::kan* | this work |
| *E. coli* |  |  |
| MM294 | *F^−^ endA-1 hsdR-1, (rk^−^, mk) supE44 thi-1 recA1* | (Backman et al., 1976) |
| DH5α | *F^−^ Φ80lacZΔM15 Δ(lacZYA-argF) U169 recA1 endA1 hsdR17 (rK^–^, mK^+^) phoA supE44 λ– thi-1 gyrA96 relA1* | Invitrogen |
| BL21(DE3) | *hsdS gal (λcts857 indt Sam7 nin5 lacUV5-T7gene* | Novagen |
| BTH101 | *F^−^cya-99 araD139 galE15 galK16 rpsL1(Str ^r^)hsdR2 mcrA1 mcrB1* | (Karimova et al., 1998) |
