# Supplementary material for: Interaction of the Morphogenic Protein RodZ with the Bacillus subtilis Min System
Source: Front Microbiol. 2018 Jan 18;8:2650. doi: 10.3389/fmicb.2017.02650 (PMC5778138; doi:10.3389/fmicb.2017.02650)
Supplement: Supplementary file 2 [file Table2.DOCX]

**Table S2. Plasmids**

| **Plasmid** | **Description** | **Reference** |
| --- | --- | --- |
| pSG1729 | *bla amyE3′ spc P_xyl_-gfp amyE5′* | (Lewis and Marston, 1999) |
| pETDuet-1 | expression vector used for protein co-expression, *bla lacI T7 promoter* | Novagen |
| pSGrodZ | *bla amyE spc P_xyl_-mgfprodZ amyE* | this work |
| pSGcyt-minJ-ypet | *bla cat cyt-minJ-ypet* | this work |
| pSGcyt-minJ-His | *bla cat cyt-minJ-His* | this work |
| pRSFDuet-1 | expression vector used for protein co-expression, *bla lacI T7 promoter* | Novagen |
| pETrodZ | *bla lacI pT7cyt-rodZ* | (Muchová et al., 2013) |
| pETminJ-S | *bla lacI pT7cyt-minJ-S* | this work |
| pETrodZminJ-S | *bla lacI pT7cyt-rodZcyt-minJ-S* | this work |
| pETminJ | *bla lacI pT7cyt-minJ* | this work |
| pKT25 | enable fusion to C-terminal end of adenylate cyclase T25 fragment | (Karimova et al., 1998) |
| pKNT25 | enable fusion to N-terminal end of adenylate cyclase T25 fragment | (Karimova et al., 1998) |
| pUT18 | enable fusion to N-terminal end of adenylate cyclase T18 fragment | (Karimova et al., 1998) |
| pUTC18 | enable fusion to C-terminal end of adenylate cyclase T18 fragment | (Karimova et al., 1998) |
| pKTrodZ | *P_lac_-T25-rodZ kan* | this work |
| pUTCrodZ | *P_lac_-T18-rodZ bla* | this work |
| pKTminJ | *P_lac_-T25-minJ kan* | R. Daniel |
| pKNTminJ | *P_lac_-minJ-T25 kan* | R. Daniel |
| pUTminJ | *P_lac_-minJ-T18 bla* | R. Daniel |
| pUTCminJ | *P_lac_-T18-minJ bla* | R. Daniel |
| pKTminC | *P_lac_-T25-minC kan* | R. Daniel |
| pKNTminC | *P_lac_-minC-T25 kan* | R. Daniel |
| pUTminC | *P_lac_-minC-T18 bla* | R. Daniel |
| pUTCminC | *P_lac_-T18-minC bla* | R. Daniel |
| pKTminD | *P_lac_-T25-minD kan* | R. Daniel |
| pKNTminD | *P_lac_-minD-T25 kan* | R. Daniel |
| pUTminD | *P_lac_-minD-T18 bla* | R. Daniel |
| pUTCminD | *P_lac_-T18-minD bla* | R. Daniel |
| pKTdivIVA | *P_lac_-T25-*d*ivIVA kan* | R. Daniel |
| pKNTdivIVA | *P_lac_-divIVA-T25 kan* | R. Daniel |
| pUTdivIVA | *P_lac_-divIVA-T18 bla* | R. Daniel |
| pUTCdivIVA | *P_lac_-T18-divIVA bla* | R. Daniel |
| pKTnoc | *P_lac_-T25-noc kan* | this work |
| pKNTnoc | *P_lac_-noc-T25 kan* | this work |
| pUTnoc | *P_lac_-noc-T18 bla* | this work |
| pUTCnoc | *P_lac_-T18-noc bla* | this work |
