# Supplementary material for: Interaction of the Morphogenic Protein RodZ with the Bacillus subtilis Min System
Source: Front Microbiol. 2018 Jan 18;8:2650. doi: 10.3389/fmicb.2017.02650 (PMC5778138; doi:10.3389/fmicb.2017.02650)
Supplement: Supplementary file 3 [file Table3.DOCX]

**Table S3. Oligonucleotides used in this work**

| **Primer** | **Sequence 5′- 3**′ |
| --- | --- |
| rodZSB | GCTGATGATGGATCCACATGACTGAACTAGGAATCCGGCTC |
| rodZEE | CATGATGCTGAATTCTTAAGATGACTTTTCTTCCTTTTTATTTACAATC |
| rodZBF | GGTAGCGCGGATCCCATGACTGAACTAGGAATCCGGCTC |
| rodZERKT | GCGGACGCTGAATTCTTAAGATGACTTTTCTTCCTTTTTATTTACAATC |
| rodZ  ER | GCGGTCCCTGAATTCGAAGATGACTTTTCTTCCTTTTTATTTACAATC |
| cminJSbg | GATGATGATAGATCTGTTAGGGCGTATTTTTCTGTC |
| ctminJEX | GATGATGATCTCGAGTGATCCCGAAGCGAC |
| cminJSB2 | GATGATGATGGATCCCATGTTAGGGCGTATTTTTCTGTCC |
| cminJEP | GATGATGATCTGCAGTTATGATCCCGAAGCGACTG |
| cminJES | CTCCTCCTCGAATTCATGTTAGGGCGTATTTTTCTGTCCATAAAG |
| cminJKE | CTCCTCCTCGGTACCTGATCCCGAAGCGACTGCTTCGTC |
| nocBF | CTACTACTAGGATCCCATGAAGCATTCATTCTCTCGTTTC |
| nocER | CTACTACTAGAATTCGATTTTGGTATGCGAATCGTTAATTG |
| nocER  KT | CTACTACTAGAATTCCTATTTTGGTATGCGAATCGTTAATTG |
| cminJEF | CGGGCCATCGAATTCATGTTAGGGCGTATTTTTCTGTCCATAAAGCAAAGAGTGAATGATAACGCGGCTCCG |
| cminJ  HisBR | AGAGCCCCCGGATCCTTATTAGTGGTGATGATGGTGATGAGATCCTGATCCCGAAGCGACTGCTTCGTCTTCACG |

The underlined areas indicate restriction sites
